# Trends in Hospital Stay, Complication Rate, and Mortality in Hip Fracture Patients: A Two-Decade Comparison at a National Tertiary Referral Center

**DOI:** 10.3390/jcm13133666

**Published:** 2024-06-24

**Authors:** Young-Seung Ko, Sang Yoon Kang, Han Jin Lee, Hong Seok Kim, Jeong Joon Yoo

**Affiliations:** 1 Department of Orthopedic Surgery, Hallym University Dong-Tan Sacred Heart Hospital, Hwaseong 18450, Republic of Korea; 2 Department of Orthopedic Surgery, Seoul National University Hospital, Seoul National University College of Medicine, Seoul 03080, Republic of Korea; 3 Department of Orthopedic Surgery, Hanil General Hospital, Seoul 01450, Republic of Korea

**Keywords:** proximal femoral fracture, length of hospital stay, complication, mortality

## Abstract

**Background**: Since the turn of the century, the age-adjusted incidence of proximal femoral fractures has caused a plateau or fall. However, it was anticipated that the number of patients with proximal femoral fractures would rise as life expectancy rose and the population over 80 years old expanded. The aim of this study was to compare the length of hospital stay, complication rate, and mortality in patients with proximal femoral fractures between two different time periods: 20 years ago and the present. **Methods**: We conducted a retrospective review of medical records of patients aged 65 years and above who underwent surgery for proximal femoral fractures between January 2000 and December 2001 and between January 2020 and December 2021. We collected information on age, gender, fracture type, length of hospital stay, and complication rate. Dates of death were obtained from the Ministry of the Interior and Safety. **Results:** We included 136 patients who were operated on between 2000 and 2001 and 134 patients between 2020 and 2021. The average age increased significantly from 71.6 years to 79.0 years (*p* < 0.001). The length of hospital stay decreased dramatically from 15.1 days to 6.0 days (*p* < 0.001). There was no statistically significant difference in delirium, urinary tract infection, or pneumonia. No difference was found in 30-day or 1-year mortality between the two groups. **Conclusions**: The complication rate and mortality between the two time periods appeared comparable, although the length of hospital stay decreased substantially. Therefore, we recommend considering expedited discharge from the acute care hospital for elderly hip fracture patients while implementing an individualized approach for better outcomes.

## 1. Introduction

It is widely known that hip fractures in the elderly have a considerable impact on morbidity, mortality, and healthcare burdens [1,2,3,4,5,6]. About half of senior patients who sustain a hip fracture are unable to achieve their prior level of mobility and independence [7,8]. Hip fractures are associated with severe morbidity, with many patients enduring a major reduction in their functional status and quality of life. Mortality rates are also notably high, with studies indicating that approximately 20–30% of patients die within the first year after a hip fracture [9,10,11]. This elevated mortality rate is often attributed to complications such as infections, cardiovascular events, and thromboembolic incidents [9]. Hip fractures also have a significant financial impact on healthcare due to the high expenses of acute hospital care, long-term rehabilitation, and the resulting rise in the requirement for long-term care facilities. The yearly direct medical expenditures associated with hip fractures are projected to be over $20 billion in the United States alone [12,13]. As the population ages and the frequency of hip fractures increases, these financial burdens are predicted to increase [14]. Recent literature reported that the age-adjusted incidence of proximal femoral fractures has resulted in a plateau or decline in absolute numbers since the turn of the century [2,8,15]. However, with an increase in life expectancy, and as the size of the population older than 80 years grows, the number of proximal femoral fracture patients is expected to increase, especially in countries entering super-aged societies [2,15,16]. It is projected that the global number of hip fractures will reach 6.3 million per year by 2050 [17,18]. Therefore, considerable interest has been shown by government and non-government healthcare agencies in changes in the treatment of proximal femoral fractures and prevention of adverse outcomes across the world [19].

The complication rate and mortality are still substantial in hip fracture patients [4,5,20]. To reduce the socioeconomic burden, the treatment of patients with proximal femoral fractures continues to evolve, with the implementation of the latest technologies to improve clinical outcomes for these patients [21]. In the same context, the length of hospital stay has also decreased over recent years, with improvements in surgical technique, multimodal analgesia, and appropriate patient education [22,23]. Some groups recently reported that delayed discharge after orthopedic surgery is associated with an increased risk of complications [24,25].The early recovery after surgery (ERAS) strategy, which integrates a multidisciplinary and multimodal approach, has been employed for patients with proximal femur fractures to optimize perioperative patient conditions and promote a prompt recovery [26,27]. However, for patients with proximal femoral fractures, days of postoperative hospitalization are required for perioperative management of chronic medical conditions.

We conducted a comparative analysis of the data from patients who underwent surgery for proximal fractures over a period of two decades to identify if there were any noticeable differences. The patients with a proximal femur fracture were of advanced age and had comorbidities, which posed difficulties in examining their long-term results. Prior research had mostly examined short-term outcomes as a result of these challenges, resulting in a lack of comprehension of long-term trends and outcomes [8,11,15]. Our objective was to analyze data from patients over a period of two decades to gain a deeper understanding of the changing patterns in patient characteristics, the presence of multiple medical conditions, and treatment outcomes. We hypothesized that patients undergoing surgery for proximal femur fractures at the present (Present group) have experienced a reduction in hospital stay duration compared to two decades earlier (Past group), while the rates of complication and mortality have not worsened. In this study, we sought to compare (1) patients’ demographic data, (2) the complication rate, and (3) the mortality rate of patients with proximal femoral fractures between two patient groups who underwent surgery for proximal fractures over a period of two decades.

## 2. Materials and Methods

### 2.1. Study Design and Participants

This study is a retrospective cohort study that included all adults aged 65 years and older who were admitted to a tertiary referral hospital in Korea for emergent femoral neck, pertrochanteric, and intertrochanteric fractures from 1 January 2000 to 31 December 2001 and from 1 January 2020 to 31 December 2021. The study population was stratified into two groups based on surgery date: (1) the Past group (January 2000 to December 2001) and (2) the Present group (January 2020 to December 2021).

The inclusion criteria were limited to patients who visited the emergency department for fresh hip fractures. The study was reviewed and approved by the Institutional Review Board of our hospital. Informed consent requirements were waived due to the retrospective nature of this study, and anonymized data were obtained from the medical records database.

Patients were excluded if they (1) were managed conservatively (non-operative), (2) were admitted primarily for other conditions apart from a hip fracture, (3) had less than one year of follow-up, or (4) were transferred to other departments for acute or chronic medical conditions.

Clinical and demographic information was collected from electronic medical records by two independent fellowship-trained orthopedic surgeons who did not participate in the initial operation. It included the patient’s age, gender, body mass index, fracture type, osteoporosis, Koval grade, comorbidities, American Society of Anesthesiology (ASA) score, hemoglobin level, admission to surgery, surgery to discharge, and the length of hospital stay. Comorbidity was measured using the updated Charlson comorbidity index (CCI) published by D’Hoore et al. [28]. The admission to surgery and the length of hospital stay were defined as the elapsed time from admission to the index operation, and from index operation to the discharge date, respectively.

### 2.2. Surgical Procedures

The surgical procedures for proximal femoral fractures encompass a range of techniques that depend on the specific fracture type and the patient’s overall health status. The primary surgical interventions conducted included total hip arthroplasty, bipolar hemiarthroplasty, internal fixation utilizing a compression hip screw, cephalomedullary nailing, multiple screw fixation, and the Femoral Neck System (DePuy Synthes, Warsaw, IN, USA). All surgeries were performed under either general, spinal, or epidural anesthesia, based on the patient’s medical condition and preferences. The option of anesthesia was determined in collaboration with the anesthesiology team to enhance patient safety and ensure maximum comfort. Preoperative management involved assessing and improving patients’ suitability for surgery, which included addressing any existing medical issues and controlling preoperative anemia. Standard surgical procedures were used throughout the intraoperative phase to reduce operative time and blood loss. Intraoperative imaging was employed to ensure the precise positioning of implants.

### 2.3. Postoperative Care

Postoperative care was standardized in order to maintain consistency and facilitate recovery. Pain was controlled using multimodal analgesia, such as patient-controlled analgesia and prophylactic antiemetic medicines, to reduce both discomfort and nausea. Immediate initiation of mobilization was advocated, commencing with the use of inclined tables and advancing to supervised walking with assistive devices, in order to avert potential problems such as deep vein thrombosis and facilitate comprehensive recuperation.

### 2.4. Complications and Mortality 

Surgical parameters assessed in this study included the type of surgery, anesthesia, time to surgery, and postoperative blood transfusion, all obtained from operative records. The occurrence of surgery-related complications and the rationale for reoperation were assessed using postoperative radiographs and medical records. Despite the existence of local complications necessitating reoperation, some patients and their families refused additional surgical intervention due to financial constraints or the patient’s overall condition. Systemic medical complications that occurred during hospitalization, including delirium, pneumonia, urinary tract infections, and deep vein thrombosis, were also obtained from electronic medical records. Mortality rates at 30 days, 180 days, and 1 year following surgery were determined using hospital records or via interviews with the patient’s family. In cases where patients were lost to follow-up, a comprehensive search for death certificates was conducted at the Ministry of the Interior and Safety.

The outcomes of interest, including surgery-related parameters and systemic complications, were compared between the two groups.

### 2.5. Statistical Analysis

The statistical significance was set at two-sided *p* < 0.05. Descriptive statistics for numerical and categorical variables were presented as means ± standard deviations and absolute values with percentages, respectively. Student’s *t*-test was used to compare continuous variables, and the chi-square test was used to compare categorical variables. In order to address any potential variables that could affect our results and ensure the accuracy of our findings, we conducted a comparison between the two groups using demographic and surgical parameters. These parameters included age, gender, BMI, fracture type, type of surgery, anesthesia, time to surgery, and postoperative blood transfusion. All analyses were performed using IBM SPSS Statistics, version 26.0 (SPSS Inc., Chicago, IL, USA).

## 3. Results

### 3.1. Characteristics of the Study Participants

**Figure 1:**
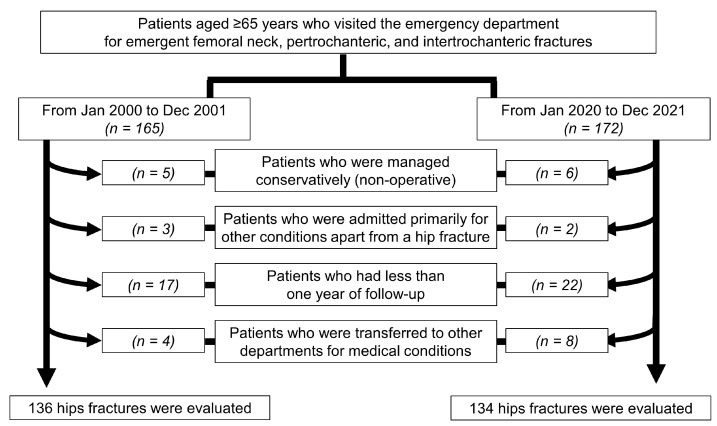
Flowchart of enrolled patients.

Between 2000 and 2001, 136 hip fractures met the study criteria, while 134 patients with hip fractures between 2020 and 2021 were included in the analysis (Figure 1). The sociodemographic characteristics of the participants are presented in Table 1.

The Present group was older than the Past cohort (71.6 ± 14.1 vs. 79.0 ± 12.0, *p*-value < 0.001). The Charlson comorbidity index of the Present group was higher than that of the Past group (4.8 ± 1.8 vs. 6.2 ± 2.2, *p*-value < 0.001), and the ambulatory status before trauma was worse in the Present group (1.5 ± 1.1 vs. 2.2 ± 1.7, *p*-value < 0.001). There was no difference in body mass index, primary diagnosis, or the proportion of osteoporosis.

### 3.2. Surgery-Related Parameters

The Present group needed more days for surgery after admission (*p* = 0.009). The proportion of total hip arthroplasty and cephalomedullary nailing increased, while that of compression hip screw treatment decreased. The proportion of patients with postoperative blood transfusion decreased (*p* < 0.001), while the average volume of transfused blood did not change much (Table 2).

### 3.3. Length of Hospital Stay, Complication Rates, and Mortality

The mean length of hospital stay was 6.0 ± 4.5 days in the Present group and 15.1 ± 7.5 days in the Past group (*p* < 0.001). There was no difference in surgery-related complications between the groups, such as periprosthetic femoral fractures (*p* = 0.282), non-union (*p* = 0.307), or reoperation (*p* = 0.777). No significant difference was found in systemic medical complications, such as delirium (*p* = 0.376), pneumonia (*p* = 0.504), deep vein thrombosis (*p* = 0.159), or acute kidney injury (*p* = 0.115). 

Difference in mortality rate between the groups was not evident: 30-day mortality (*p* = 0.121), 180-day mortality (*p* = 0.147), and one-year mortality (*p* = 0.054) (Table 3).

## 4. Discussion

This observational retrospective study investigated changes in the length of hospital stay, complication rates, and mortality of hip fracture patients over a period of two decades. Our study revealed significant differences between the two groups in mean age, which rose from 71.6 to 79.0, as well as in the Charlson comorbidity index and pre-trauma Koval grade. Our findings indicated a significant decrease in the length of hospital stay, from 15.1 days to 6.0 days. However, there were no significant variations observed in the rates of complications and mortality between the two cohorts. 

### 4.1. Age and Comorbidity

In our study, the average age of patients who underwent surgery for proximal femur fractures showed a substantial increase, rising from 71.6 years in the Past group to 79.0 years in the Present group. According to recent studies, the age-adjusted incidence of proximal femoral fractures has either plateaued or decreased in absolute numbers [2,8,15]. Nonetheless, the number of proximal femoral fracture patients is anticipated to rise as life expectancy rises and the senior population over 80 years old develops [2,15,16]. These trends are predicted to intensify in countries moving toward super-aged societies, with a notable rise in the number of senior patients in need of hip fracture surgery [11,15]. Moreover, the increase in average age contributed to a higher Charlson comorbidity index (CCI) and a higher pre-trauma Koval grade in the Present group. The Present group had a significantly higher CCI (6.2 vs. 4.8, *p* < 0.001) than the Past group, which suggested that the older cohort had a larger burden of comorbid conditions [15]. Furthermore, the Present group’s higher Koval grade indicated a worse pre-trauma ambulatory state, which was probably caused by the concomitant impact of age and comorbid diseases [9,11]. These results highlighted the need for perioperative care techniques that were specifically designed to meet the complicated health profiles of older hip fracture patients in order to ensure optimal outcomes for this expanding population.

### 4.2. Changes in Perioperative Protocols in the Ward

Along with preoperative optimization, current postoperative care in the ward for geriatric hip fracture patients has dramatically changed from the past and is crucial to ensure optimal outcomes; this comprehensive perioperative care is referred to as early recovery after surgery (ERAS) [27,29]. ERAS involves a multimodal approach to perioperative care, aimed at minimizing surgical stress, preserving postoperative physiological function, and accelerating recovery. The essential elements of ERAS protocols include preoperative counseling, optimized anesthesia and analgesia, minimal use of drains and tubes, early mobilization, and a structured approach to nutrition. Preoperatively, patients received thorough education about the surgical process and postoperative expectations, which helped to reduce anxiety and improve compliance. Intraoperatively, techniques such as regional anesthesia and minimally invasive surgical methods were employed to minimize surgical trauma and pain. Postoperatively, early mobilization and physical therapy were prioritized to promote quicker functional recovery, reduce the risk of complications such as deep vein thrombosis, and decrease the length of hospital stay. Studies demonstrated that ERAS reduced the length of hospital stay, lowered complication rates, and enhanced overall patient satisfaction [30,31]. In our hospital, wound drainage has not been used since 2016, and a patient blood management protocol has been established. This protocol involves not only monitoring hemoglobin levels but also identifying and treating the cause of anemia [32]. Moreover, the early initiation of rehabilitation is critical in promoting a return to pre-injury daily activities. Effective management of postoperative pain using patient-controlled anesthesia and prophylactic antiemetic medications is essential to minimize patient discomfort, prevent postoperative nausea and vomiting, and facilitate early mobilization. Finally, the implementation of an early functional rehabilitation protocol, starting from the use of tilting tables to assisted gait with ambulatory devices, is another factor in preventing complications such as deep vein thrombosis and promoting mobility and overall recovery, and in decreasing the length of hospital stay.

### 4.3. Admission to Surgery and Length of Hospital Stay

Determining the optimal surgical time for elderly hip fracture patients still remains controversial in daily practice [33,34,35]. Previously, Kjaervik et al. conducted a large cohort study using the Norwegian Hip Fracture Register and reported that prolonged time to surgery was associated with increased 30-day and one-year mortality rates [36]. Furthermore, other studies concluded that not receiving expedited surgery is associated with a higher risk for pneumonia, pressure ulcers, delirium, an increased length of hospital stay, and even higher mortality [37,38]. In our data, the time to surgery was different between the Past group and the Present group (2.0 vs. 2.8, *p* < 0.05), with comparable mortality rates. The difference in the time to surgery in our study could be due to the difference in the preoperative CCI score, as in the study of Kjaervik et al. where patients with a longer time to surgery have higher CCI scores [36]. 

Controversy also exists over the length of hospital stay after the operation. The concept that a reduction in the length of hospital stay would contribute to lowering the complication rate and mortality rate has been discussed recently. The COVID-19 pandemic accelerated this major transition, especially in total joint arthroplasty, notably with the increased utilization of same-day discharge pathways, without increasing readmissions or reoperations [39]. Certainly, the surgery and the length of hospital stay of emergent hip fracture patients differ from elective total joint arthroplasties, but an expedited discharge program has recently been started on hip fracture patients. Introducing a clinical pathway, the Queen Mary Hospital in Hong Kong decreased their 30-day mortality rates for elderly hip fracture patients to 1.7% in 2009 [40]. In our hospital, a routine clinical pathway was performed on every hip fracture patient, and the length of hospital stay then decreased dramatically over two decades, from 15.1 to 6.0 days (*p* < 0.001), without increasing the complication and mortality rates. Early discharge from the acute care hospital may provide the same outcome without increasing morbidity and mortality in hip fracture patients, decreasing the socioeconomic burden.

### 4.4. Complication Rates

In our study, the complication rate was comparable between the Past and Present groups. Considering that the patients of the Present group were older and the CCI higher, the result might seem encouraging. This result might also be affected by selection bias. We excluded the patients who were transferred to other departments after surgery due to the management of medical comorbidities. These patients might possess more complications than the studied subjects. 

Moreover, another reason for the similar complication rate between the two cohorts might be related to transfusion. The proportion of patients who received blood transfusion was lower in the Present group (88.2% vs. 41.8%). The average volume of transfusion, however, was comparable. In our hospital, we abandoned the routine use of the wound drainage system from 2016 and adopted a strict patient blood management protocol as previously described [32]. A number of studies reported reduced blood use with similar or improved clinical outcomes in the orthogeriatric care service as well as in general orthopedic surgery [41,42].

### 4.5. Mortality

In our analysis, we found that 30-day, 180-day, and 1-year mortality in our hip fracture patients was not significantly different between the two time periods. These rates were compatible with or slightly lower than the findings of previous studies [5,43]. One of the reasons for our non-significant difference between groups was due to the graveness of proximal femoral fractures, regardless of the improvement of perioperative management. Though statistically non-significant, however, the patients in the Present group showed higher mortality. As mentioned previously, the patients of the Present group tended to be older, and it is widely known that increasing age was associated with a higher risk of mortality across previous studies [44,45].

However, there were some limitations in this study. Firstly, due to the study’s retrospective design, the risk of selection bias in data cannot be avoided. Significant differences were observed between the two groups in terms of age and CCI, whereas no disparities were discovered in gender, BMI, diagnosis, osteoporosis, or hemoglobin level. This might be attributed to the growing prevalence of proximal femur fractures in elderly patients over the last twenty years, which aligns with the overall rise in average life expectancy. Furthermore, we were unable to do a multivariable analysis to account for variations in demography and healthcare practices across the two-decade period. Our analysis may be affected by confounding variables that were not accounted for, thereby limiting the reliability of our findings. However, future research could benefit from including a subset analysis of conservatively managed patients to compare outcomes comprehensively. In addition, we excluded patients who had non-operative treatment for their proximal femoral fractures. Non-operative cases were omitted due to their distinct patient profiles and treatment regimens, which varied from those of surgical cases. Including non-operative patients might have resulted in substantial variability and confounded the study outcomes. Moreover, this study was performed in a single tertiary referral hospital in an Asian country with a relatively small number of patients; therefore, generalization of our findings to other countries must be cautious. Moreover, the complication rate might be underreported and underestimated since some patients or family members might refuse to undergo further management because of poor general conditions or financial problems. To further validate our findings and improve their generalizability, we suggest that future multicenter studies and meta-analyses be conducted. In an effort to develop a more comprehensive understanding of the trends and outcomes associated with hip fracture treatment across various regions and healthcare systems, these studies should endeavor to incorporate a broader range of populations and healthcare settings.

## 5. Conclusions

We found a decreased length of hospital stay with a comparable rate of complications and mortality for elderly hip fracture patients in two separate periods: twenty years ago and the present. Given the increase in the older adult population with increased osteoporosis, sarcopenia, frailty, and related fracture risks, these findings have considerable implications for the legislation of healthcare policy and the development of clinical pathways. To further validate our findings and enhance their generalizability, we suggest conducting future multicenter studies and meta-analyses across more diverse populations and healthcare settings.

## Figures and Tables

**Table 1:** Baseline characteristics of the participants.

	Past Group	Present Group	*p*-Value
Number	136	134	
Age (years)	71.6 ± 14.1 (95% CI * 69.2–74.0)	79.0 ± 12.0 (95% CI 77.0–81.0)	<0.001
Female sex, n (%)	93 (68.4%)	93 (69.4%)	0.856
Body mass index (kg/m^2^)	22.4 ± 4.2	23.6 ± 3.7	0.270
Diagnosis			0.121
Neck fracture, n (%)	72 (52.9%)	82 (61.2%)	
Intertrochanteric fracture, n (%)	64 (47.1%)	52 (38.8%)	
Osteoporosis (%)	60/91 (65.9%)	86/120 (71.7%)	0.372
Charlson comorbidity index	4.8 ± 1.8 (95% CI * 4.5–5.1)	6.2 ± 2.2 (95% CI * 4.0–8.4)	<0.001
Pre-trauma Koval grade	1.5 ± 1.1	2.2 ± 1.7	<0.001
Preoperative hemoglobin	11.9 ± 2.0	12.1 ± 2.0	0.404

* CI: confidence interval.

**Table 2:** Comparison of surgery-related parameters between hip fracture patients of the present and of twenty years ago.

	Past Group (n = 136)	Present Group (n = 134)	*p*-Value
Admission to surgery (days)	2.0 ± 1.5	2.8 ± 3.0	0.009
Name of the operation, n (%)			
Total hip arthroplasty	2 (1.5%)	17 (12.7%)	
Bipolar hemiarthroplasty	58 (42.6%)	57 (42.5%)	
Internal fixation using			
Compression hip screw	63 (46.3%)	5 (3.7%)	
Cephalomedullary nailing	3 (2.2%)	46 (34.3%)	
Multiple screw fixation	10 (7.4%)	5 (3.7%)	
Femoral Neck System *	0 (0.0%)	4 (3.0%)	
Anesthesia, n (%)			0.021
General	18 (13.2%)	28 (20.9%)	
Spinal	110 (80.9%)	105 (78.4%)	
Epidural	8 (5.9%)	1 (0.7%)	
Transfusion, n (%)	120 (88.2%)	56 (41.8%)	<0.001
Average transfusion volume (mL)	833.9 ± 843	746.7 ± 610.8	0.487

* Femoral Neck System (DePuy Synthes, Warsaw, IN, USA).

**Table 3:** Comparison of length of hospital stay, complication rates, and mortality rate between hip fracture patients of the present and twenty years ago.

	Past Group (n = 136)	Present Group (n = 134)	*p*-Value
Number	136	134	
Length of hospital stay (days)	15.1 ± 7.5 (95% CI * 13.9–16.4)	6.0 ± 4.5 (95% CI * 5.2–6.8)	<0.001
Surgery			
Total hip arthroplasty	16.0 ± 2.8	4.5 ± 0.9	0.107
Bipolar hemiarthroplasty	15.2 ± 7.6	4.4 ± 0.6	<0.001
Internal fixation using			
Compression hip screw	15.1 ± 7.5	12.2 ± 5.5	0.400
Cephalomedullary nailing	25.7 ± 7.2	6.1 ± 4.8	<0.001
Multiple screw fixation	10.2 ± 4.2	8.6 ± 7.4	0.596
Femoral Neck System **	-	4.0 ± 1.4	-
Surgery-related complications, n (%)			
Periprosthetic fracture	6 (4.4%)	2 (1.5%)	0.282
Non-union	1 (0.7%)	3 (2.2%)	0.307
Dislocation	0 (0.0%)	0 (0.0%)	-
Infection	0 (0.0%)	0 (0.0%)	-
Screw cut-out	2 (1.5%)	0 (0.0%)	0.159
Reoperation	6 (4.4%)	5 (3.7%)	0.777
Medical complications, n (%)			
Delirium	19 (14.0%)	24 (17.9%)	0.376
Pneumonia	4 (2.9%)	6 (4.5%)	0.504
Urinary tract infection	1 (0.7%)	6 (4.5%)	0.053
Deep vein thrombosis	2 (1.5%)	0 (0.0%)	0.159
Voiding difficulty	12 (8.8%)	8 (6.0%)	0.371
Acute kidney injury	5 (3.7%)	11 (8.2%)	0.115
Follow-up duration (years)	6.0 ± 6.8	1.3 ± 0.9	<0.001
Mortality, n (%)			
30-day mortality	0 (0.0%)	3 (2.2%)	0.121
180-day mortality	4 (2.9%)	9 (6.7%)	0.147
1-year mortality	11 (8.1%)	21 (15.7%)	0.054

* CI: confidence interval; ** Femoral Neck System (DePuy Synthes).

## Data Availability

The dataset supporting the conclusions of this article is included within the article.
